# Exposures to Air Pollutants during Pregnancy and Preterm Delivery

**DOI:** 10.1289/ehp.8733

**Published:** 2006-02-16

**Authors:** Jong-Han Leem, Brian M. Kaplan, Youn K. Shim, Hana R. Pohl, Carol A. Gotway, Stevan M. Bullard, J. Felix Rogers, Melissa M. Smith, Carolyn A. Tylenda

**Affiliations:** 1 Department of Occupational and Environmental Medicine, Inha University, Incheon, Korea; 2 Division of Toxicology and Environmental Medicine and; 3 Division of Health Studies, Agency for Toxic Substances and Disease Registry, Atlanta, Georgia, USA; 4 Division of Environmental Hazards and Health Effects Biometry Activity, National Center for Environmental Health and; 5 Office of Science, Office of the Director, National Immunization Program, Centers for Disease Control and Prevention, Atlanta, Georgia, USA

**Keywords:** adverse birth outcomes, exposure, geographic information system, GIS, Korea, kriging methods, risk assessment, susceptibility

## Abstract

The association between preterm delivery (PTD) and exposure to air pollutants has recently become a major concern. We investigated this relationship in Incheon, Republic of Korea, using spatial and temporal modeling to better infer individual exposures. The birth cohort consisted of 52,113 singleton births in 2001–2002, and data included residential address, gestational age, sex, birth date and order, and parental age and education. We used a geographic information system and kriging methods to construct spatial and temporal exposure models. Associations between exposure and PTD were evaluated using univariate and multivariate log-binomial regressions. Given the gestational age, birth date, and the mother’s residential address, we estimated each mother’s potential exposure to air pollutants during critical periods of the pregnancy. The adjusted risk ratios for PTD in the highest quartiles of the first trimester exposure were 1.26 [95% confidence interval (CI), 1.11–1.44] for carbon monoxide, 1.27 (95% CI, 1.04–1.56) for particulate matter with aerodynamic diameter ≤ 10 μm, 1.24 (95% CI, 1.09–1.41) for nitrogen dioxide, and 1.21 (95% CI, 1.04–1.42) for sulfur dioxide. The relationships between PTD and exposures to CO, NO_2_, and SO_2_ were dose dependent (*p* < 0.001, *p* < 0.02, *p* < 0.02, respectively). In addition, the results of our study indicated a significant association between air pollution and PTD during the third trimester of pregnancy. In conclusion, our study showed that relatively low concentrations of air pollution under current air quality standards during pregnancy may contribute to an increased risk of PTD. A biologic mechanism through increased prostaglandin levels that are triggered by inflammatory mediators during exposure periods is discussed.

Preterm delivery (PTD) remains the leading cause of perinatal mortality and occurs in approximately 4–10% of pregnancies (Leem et al. 2002; Reagan and Salsberry 2005). Known risk factors for PTD include lower social class, less education, single marital status, low income, younger maternal age, low body weight, ethnicity, smoking, and poor housing, along with medical factors such as induction, premature rupture of membranes, infection, multiple pregnancy, intrauterine death, fetal and uterine abnormalities, and chorioamnionitis (Bibby and Stewart 2004). Associations between ambient air pollutants and adverse pregnancy outcomes have also been reported (Chen et al. 2002; Ha et al. 2001; Lin et al. 2001; Maisonet et al. 2001, 2004; Maroziene and Grazuleviciene 2002; Šrám et al. 2005; Wilhelm and Ritz 2003). The ambient air pollutants of concern in these studies include carbon monoxide, nitrogen dioxide, sulfur dioxide, ozone, and particulate matter (PM). Because of differences in pathogenic mechanisms, the effects of air pollutants in these studies were analyzed separately for each perinatal outcome. Additional studies have reported an association between exposure to air pollutants during critical periods of pregnancy and PTD (Bobak 2000; Liu et al 2003; Mohorovic 2004; Ritz et al. 2000; Tsai et al. 2003; Woodruff et al. 2003; Xu et al. 1995; Yang et al. 2002a, 2002b, 2003, 2004), although the biologic mechanism that mediates the link between exposure to air pollutants and PTD is not well understood. Previous studies for adverse pregnancy outcomes, however, had limited spatial and temporal information on pollution sources and concentrations.

The purpose of this study was to investigate the associations between air pollution and PTD in Incheon, Republic of Korea (Korea). The study has two main objectives. The first is to construct spatially and temporally explicit surfaces of atmospheric pollutants that serve as surrogates for potential exposure to air pollution, corresponding to the first, second, and third trimesters of pregnancy. A second, and the primary, objective of this study is to relate these exposure surfaces to PTD. The results of this study will provide a greater understanding of the effect of air pollution on PTD and the impact of potential exposure on critical periods of pregnancy, and suggest possible hypotheses about the biologic mechanism linking exposure to air pollutants and PTD.

## Materials and Methods

### Study population

We examined 53,514 singleton birth records with birth dates from 1 January 2001 to 31 December 2002 in Incheon, Korea. The records were obtained from the Korean National Birth Register (Daejeon, Korea), complying with the applicable human subject research requirements of the authors’ institutions. In Korea, physicians or nurses complete birth certificates at delivery and register them with regional birth registries. From the register, we obtained information on whether or not a birth was a PTD, defined as a live birth < 37 weeks of gestation. We also obtained information on maternal residential address at the time of birth, gestational age, birth date, infant sex, birth order, and parental age, occupation, and education. Gestational age was determined by the responsible obstetrician, based on all available information, including date of last menstrual period and the mother’s estimate of the date of conception. In recent years, the use of ultrasound dating for the determination of gestational age has been increasing in Korea. We excluded 247 deliveries with missing values on parental age, parental education level, or gestational age. Because valid exposure data were not available on islands near Incheon, we also excluded 1,154 deliveries from that area in the final analysis. Therefore, this study included a total of 52,113 deliveries. Each mother’s residential address at the time of child’s birth included assignment to one of 132 administrative units called dongs. In urban areas, the dong typically encompasses a few city blocks, whereas in rural areas, the dong is roughly equivalent to a U.S. county. For comparison, the mean area of a dong is 7.82 km^2^ (median, 1.42 km^2^), whereas the mean area of a U.S. ZIP code is 296.59 km^2^ (median, 98.82 km^2^). The exclusion of the islands in this study reduced the number of dongs to 120.

### Air pollution data and spatial mapping

**Figure 1:**
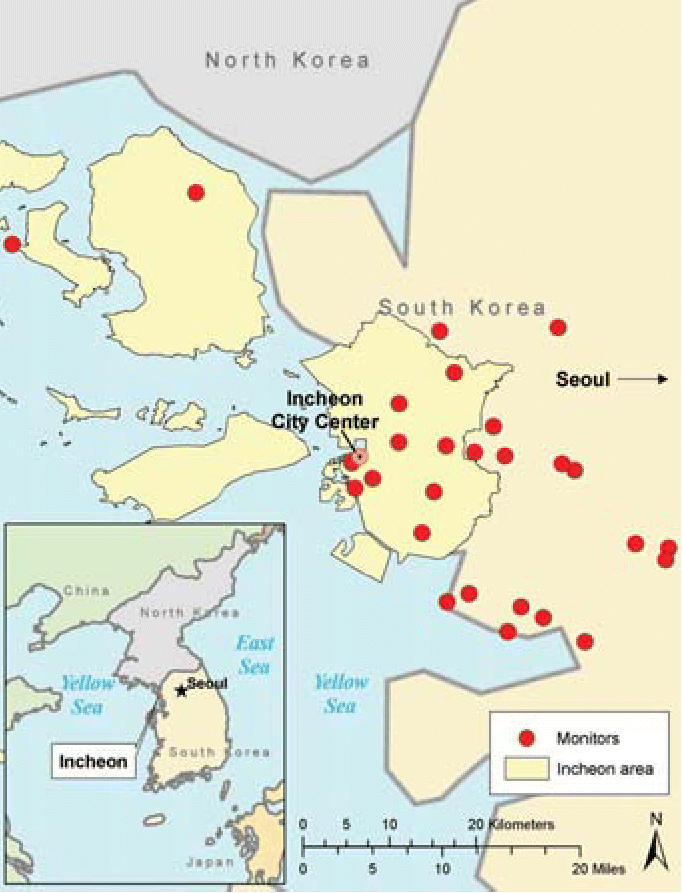
Air monitoring stations near Incheon.

Measurements of air pollutants were compiled from air monitoring data routinely collected at 11 monitoring stations in Incheon and 16 monitoring stations located in the areas surrounding Incheon (Figure 1). These air monitoring stations are located around industrial and residential areas as well as in regions where air pollution levels are low. For each monitoring station, the data include 1-hr concentrations of the gaseous pollutants SO_2_, NO_2_, CO, and ambient PM with aerodynamic diameter ≤10 μm (PM_10_). Briefly, SO_2_ was measured by pulse ultraviolet fluorescence, NO_2_ by chemiluminescent methods, CO by nondispersive infrared methods, and PM_10_ by beta-ray absorption methods (National Institute of Environmental Research 2003). The hourly data available for gaseous pollutant and PM_10_ in each monitoring stations were obtained from the Korean National Institute of Environmental Research (2005) and used to determine daily and monthly averages.

**Figure 2:**
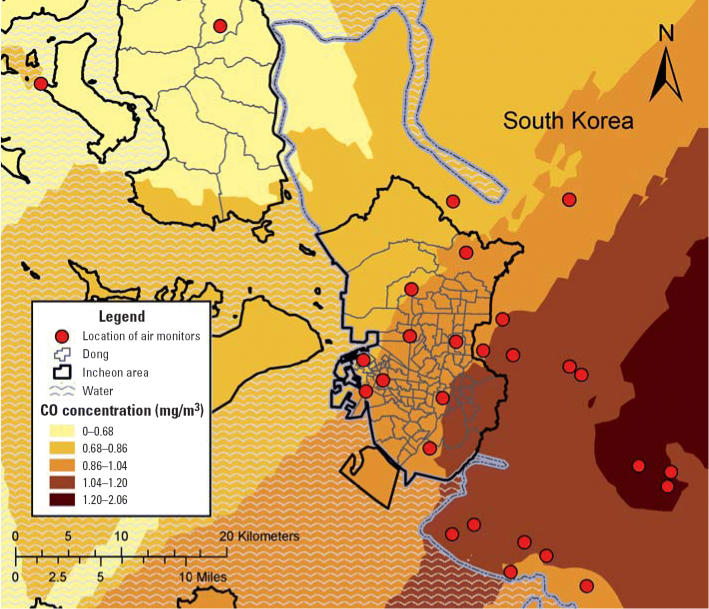
An example of estimated air pollution levels using air monitoring data and kriging in the metropolitan area of Incheon, South Korea.

Pollutant levels for each dong by month from April 2000 through December 2002 for SO_2_, NO_2_, CO, and PM_10_ were predicted from the levels recorded at the monitors using a method known as ordinary block kriging, which was implemented with the Geostatistical Analyst extension of ArcGIS (ArcMap, version 9.0; ESRI Inc., Redlands, WA, USA) using 0.170 km × 0.170 km grids to partition each dong for each pollutant and each month. Block kriging is a statistical mapping technique that allows the prediction of an average concentration over a spatial region from data collected at point locations (Isaaks and Srivastava 1989; Waller and Gotway 2004). Kriging involves estimating a smooth surface from data points over the domain. Predictions from block kriging are based on kriging using a regular grid and then averaging the values within each block. In this case, the blocks are the 120 dongs. Figure 2 shows an example of the spatial trend of CO for January 2002 predicted with ordinary kriging using the 27 monitors. The average pollutant concentration for each dong predicted from block kriging was used in the subsequent calculations.

**Figure 3:**
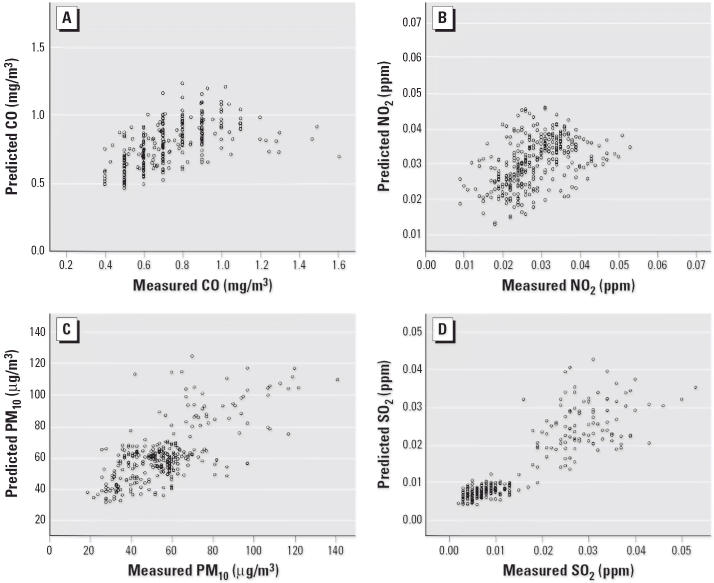
Plots of observed values at air monitoring stations and predicted values by the kriging method.

We evaluated the quality of the predicted values from kriging using cross-validation, a technique with which each monitoring station is removed, one at a time, and the concentration at each omitted station is predicted using the concentration values observed at the other monitors. The observed (measured) concentrations at the ambient monitoring sites were then compared with the values predicted by kriging (Figure 3).

### Exposure assessment

We used residential addresses at the time of birth for spatial exposure assignment. We assigned air pollutant concentrations for each trimester of the pregnancy based on location of the residence at the time of birth; the study population was assumed to be stable (did not move) over the exposure time period. The monthly average pollutant concentration in each dong was matched temporally to the date of birth and length of gestation. For each live birth, therefore, average air pollution concentrations were retrospectively calculated for the first, second, and third trimester. For PTD, the potential exposure window was expressed in trimesters.

### Statistical analysis

The fundamental hypothesis in this study is that the temporal and spatial variation in ambient air pollution levels is associated with temporal and spatial variation in PTD. PTD was defined dichotomously and served as the dependent variable in the analysis. Average daily concentrations of ambient SO_2_, NO_2_, CO, and PM_10_ represent the independent variables. The quartiles of the distribution of the concentration values for each pollutant in the controls were used to assign relative exposure categories. We examined the associations between the individual-level dependent variable and independent variables by univariate and multivariate log-binomial regressions (Wacholder 1986) corrected for overdispersion. The log link function was used instead of the traditional logit link function to obtain estimates of risk ratio (RR) instead of odds ratios. We calculated adjusted RRs and 95% confidence intervals (CIs) for PTD in relation to exposure to ambient air pollutants after controlling for the effects of maternal age (< 20, 20–24, 25–29, ≥30 years), parity, sex, season of birth, and education level of each parent. We used the likelihood ratio test to examine the statistical significance of the dose–response relationships between the air pollutants and PTD risk.

## Results

We observed statistically significant positive correlations among SO_2_, NO_2_, and CO, with coefficients ranging from 0.31 to 0.63 (Table 1). However, PM_10_ had generally weak positive associations with NO_2_, CO, and SO_2_, with coefficients of 0.37, 0.27, and 0.13, respectively.

Crude RRs with 95% CIs for the potential confounding factors for PTD are shown in Table 2. The RRs for PTD were increased for the following risk factors: maternal age < 20 years, maternal age ≥30 years, mother’s education > 16 years, father’s education < 16 years, and season (January to February 2001 and June to August 2002). We evaluated the quality of the predicted values from the kriging technique using cross-validation for CO, PM_10_, SO_2_, and NO_2_ (Figure 3). The geometric means of predicted/observed values are 1.07, 1.10, 1.04, and 0.96 for CO, PM_10_, NO_2_, and SO_2_, respectively, indicating that the kriging technique produced reasonable results. Correlation coefficients between predicted values and observed values for PM_10_, SO_2_, CO, and NO_2_ are 0.75, 0.83, 0.45, and 0.61, respectively. Table 3 shows the crude and adjusted RRs with their 95% CIs of PTD for maternal potential exposure to CO, PM_10_, NO_2_, and SO_2_ during the first trimester of pregnancy. The crude RR for PTD with potential exposure to CO at the highest quartile (0.91–1.27 μg/m^3^) during the first trimester was 1.20 (95% CI, 1.06–1.34), compared with the lowest quartile (0.47–0.63 μg/m^3^). The adjusted RR for PTD with potential exposure to CO at the highest quartile during the first trimester was 1.26 (95% CI, 1.11–1.44). The dose–response relationship between CO exposure and PTD was significant (*p* < 0.001). Similar increases were observed for the highest quartiles of NO_2_ (adjusted RR = 1.24; 95% CI, 1.09–1.41), PM_10_ (adjusted RR = 1.27; 95% CI, 1.04–1.56), and SO_2_ (adjusted RR = 1.21; 95% CI, 1.04–1.42), compared with the lowest quartiles. The dose–response relationships between PTD and exposures to NO_2_ and SO_2_ were statistically significant (*p* = 0.02 and *p* = 0.02, respectively). Table 4 shows the crude and adjusted RRs of PTD for maternal exposures to PM_10_, CO, SO_2_, and NO_2_ during the third trimester. The RRs were increased for the highest quartiles of CO (adjusted RR = 1.16; 95% CI, 1.01–1.34) and NO_2_ (adjusted RR = 1.21; 95% CI, 1.07–1.37), compared with the lowest quartiles, during the third trimester. The dose–response relationship was significant for both CO (*p* = 0.03) and NO_2_ (*p* < 0.001) exposures during the third trimesters.

There appears to be a general air pollution effect within more than one window of susceptibility with a more pronounced effect during the first trimester. When adjusted RRs were compared with crude RRs, the RR for PM_10_ at the highest quartile showed the greatest increase, 19%. The RRs for the other pollutants showed less than a 10% increase after controlling for confounders.

## Discussion

In our study, the highest ambient air pollution concentrations during the first trimester were significantly associated with elevated relative risks of PTD. Similar results were found for NO_2_ and CO during the third trimester. These results are generally consistent with the findings from China, the United States, Canada, and the Czech Republic (Bobak 2000; Liu et al. 2003; Mohorovic 2004; Ritz et al. 2000; Tsai et al. 2003; Woodruff et al. 2003; Xu et al. 1995; Yang et al. 2002a, 2002b, 2003, 2004). These studies reported significant associations between air pollution and PTD during early pregnancy (i.e., first or second month, first trimester) (Mohorovic 2004; Ritz et al. 2000), late pregnancy (i.e., last month, last trimester, 7 days or 6 weeks before birth) (Liu et al. 2003; Xu et al. 1995), or during both early and late pregnancies (Bobak 2000).

Our study has several strengths. First, this birth cohort study is population based and is less likely to suffer from selection bias than other studies. Second, the present study is one of only a few studies using a large sample size to assess the potential effects of maternal exposure to ambient air pollutants on PTD. A larger cohort size might have further improved this study; however, when this study was initiated, the 2003 birth cohort data were not available and the data before 2001 did not contain residential addresses. Third, birth records in Korea are generally accepted as complete, with reliable individual information on both parents and infants recorded on each certificate. Therefore, we were able to estimate the risks after controlling for the effects of potential confounding factors.

Finally, a more accurate exposure assessment for individual mothers was carried out in our study. Reliable measurements of daily SO_2_, NO_2_, CO, and PM_10_ concentrations were available from several air monitoring stations throughout Incheon, and our study used kriging methods to predict the spatial distribution of the air pollutants (Jerrett et al. 2005b; Mulholland et al. 1998; Pikhart et al. 2001). The kriging method, unlike proximity models (Jerrett et al. 2005a), uses real pollution measurements in the computation of exposure estimates. In situations where many monitoring stations exist, kriging methods are often preferred to other interpolation methods because they are fairly accurate in a variety of situations and avoid the artifacts that often result from the use of inverse distance weighted, spline, or global/local polynomials (Jerrett et al. 2005a; Ritz et al. 2000; Waller and Gotway, 2004). Therefore, our assignment of exposure using monthly block kriging from air monitoring stations is one of the preferred methods.

This study also has several weaknesses. First, maternal smoking and environmental tobacco smoke are well-known risk factors for adverse pregnancy outcomes, but this information was not available from the birth registry. However, because most women in Korea are not likely to smoke during pregnancy (Korean National Statistical Office 2005; Pritham and Sammons 1993), omission of this risk factor from the analyses is not likely to bias the results. Second, although our study attempted to decrease misclassification of individual exposures by enhancing exposure assessment through spatially and temporally explicit exposure models, the potential for misclassification of exposure due to the use of surrogate ambient air pollution data still exists. The only real way to avoid such potential misclassifications is to conduct personal exposure assessments, which are often not feasible. Third, although we had access to a relatively high density of 27 air monitoring stations near and around Incheon and used block kriging to construct spatial exposure surfaces, the uncertainty of the predicted average concentrations for the dongs was not incorporated into the regression analyses. This is a common limitation of nearly all similar studies because error propagation is computationally difficult. Finally, because we could not geocode the residential addresses to point locations, the analysis is “ecologic,” meaning that the results associated with the dong level may not apply to individuals and that an analysis using different administrative units could produce different results.

Several hypotheses have been postulated to explain the mechanism of triggering PTD. One hypothesis suggests causality between uterine inflammation and PTD. The direct evidence that infection provokes preterm labor was first shown in an animal study. When group B streptococci were injected into the amniotic fluid in preterm rhesus monkeys, amniotic fluid cytokine concentrations increased, followed by production of the prostaglandins E_2_ and F_2α_, and finally uterine contractions (Gravett et al. 1994). Similarly, in humans, preterm labor due to infection is thought to be initiated by cytokines, including interleukin-(IL)1, tumor necrosis factor, and IL-6, produced by macrophages (Cram et al. 2002; Mitreski and Radeka 2002; Narahara and Johnston 1993).

Because IL-1β is not present in the membranes of term-laboring patients, it may be the unique mediator by which intrauterine infection induces preterm labor (Cunningham and William 1997). Antenatal infection can trigger intrauterine inflammation, which then promotes preterm labor. In addition, periodontal disease may be an independent risk factor for preterm labor: Postulated mechanisms include translocation of periodontal pathogens to the fetoplacental unit and action of a periodontal reservoir of lipopolysaccharides or inflammatory mediators (McGaw 2002). Our inability to determine periodontal status of the mother is a potential confounding factor. Cyclooxygenase-2 inhibitor, developed as an anti-inflammatory drug, also has toxolytic effects (Sakai et al. 2001). A similar inflammatory mechanism has been suggested for the effect of smoking on fetal growth retardation, PTD, and perinatal mortality (Klesges et al. 2001). There are reports of increased blood viscosity and plasma fibrinogen during air pollution (Peters et al. 1997). It has been speculated that chronic exposure to high pollution levels may influence placental function (Petruzzelli et al. 1998). The placental dysfunction may lead to intrauterine fetal growth retardation. The effects of air pollution on pregnancy outcomes may differ with the timing of exposure, with early exposures likely to be important for pregnancy end points such as spontaneous abortion, intrauterine growth retardation, and birth defects (Antipenko and Kogut 1993; Dejmek et al. 1999, 2000; Hansteen et al. 1987). Intrauterine infection during pregnancy could also lead to brain damage of the developing fetus (Huleihel et al. 2004).

Recent studies suggest that antenatal infection and inflammation can increase the preterm infant’s susceptibility to develop chronic lung disease. It may be that exposure of the fetal lung to high concentrations of proinflammatory cytokines is the cause of this increased susceptibility (Miralles et al. 2002). Photochemically produced gaseous products influence the toxic responses of cells, such as production of cytokines, in the absence of particles (Sexton et al. 2004). PM_10_ is responsible for the production and the release of inflammatory cytokines by the respiratory tract epithelium as well as for activation of the transcription factor nuclear factor κB (Baeza-Squiban et al. 1999; Bonvallot et al. 2001). Although fetal exposures to air pollution are probably much lower than exposure to the constituents of cigarette smoke, we propose that the biologic mechanism of PTD could be through increased prostaglandin levels that are triggered by inflammatory mediators during exposure periods.

The pathophysiology of CO may be more complex, involving hypoxic stress on the basis of interference with oxygen transport to the cells and possibly impairment of electron transport. CO can also affect leukocytes, platelets, and the endothelium, inducing a cascade of effects resulting in oxidative injury (Hardy and Thom 1994). CO may interfere with metabolic and transport function of the placenta and, after crossing the placental barrier, concentrate more in the fetus than in the mother (Hardy and Thom 1994). Neonates and fetuses are more vulnerable because of the natural leftward shift of the dissociation curve of fetal hemoglobin, a lower baseline P_O_2__ (partial pressure of oxygen), and carboxyhemoglobin levels at equilibration that are 10–15% higher than maternal levels (Jaeger et al. 2000).

The causality between air pollution and risk of intrauterine growth retardation and decreased birth weight, birth length, and head circumference has been suggested through molecular epidemiologic studies where levels of DNA adducts are positively correlated with these outcomes (Šrám et al. 2005). The DNA damage may occur through exposure to poly-cyclic aromatic hydrocarbons. Although this study identifies an association between air pollution and PTD, PTD may be less sensitive to air pollution, possibly because of the postulated multifactorial nature of this health outcome.

In this study, we observed air pollution levels critical to PTD in humans. These levels are important because they may be a good indication on how to protect fetuses against adverse effects from air pollutants. In Korea, the current annual air quality standards are 52.4 μg/m^3^ for SO_2_, 94 μg/m^3^ for NO_2_, and 70 μg/m^3^ for PM_10_. The CO standard over 8 hr is 10.4 mg/m^3^. Korea’s annual standard for air quality is certainly too high and does not prevent adverse pregnancy outcomes. Our study showed that statistically significant effects of PTD are seen below the air quality standards for CO and NO_2_ and potentially below the standards for PM_10_ and SO_2_. Our study may provide supportive evidence that reduction in the current air quality standards may improve pregnancy outcomes.

In conclusion, our study showed that relatively low concentrations of air pollution under current air quality standards during critical gestational periods may contribute to increased risk of PTD. Our results also suggest that fetuses in the early and late stages of development are susceptible to air pollutants. Further studies are needed to validate fetal susceptibility to air pollutants with more detailed information on personal exposures, confounders, and effect modifiers.

## Figures and Tables

**Table 1:** Pearson correlation coefficients among daily average concentrations of PM_10_ and gas pollutants, Incheon, 2001–2002.

	PM_10_	CO	NO_2_	SO_2_
PM_10_	1.00			
CO	0.27*	1.00		
NO_2_	0.37*	0.63*	1.00	
SO_2_	0.13*	0.31*	0.54*	1.00

* *p*-Value < 0.001.

**Table 2:** Crude RRs (95% CIs) of potential confounding factors for PTD.

	Cases (*n* = 2,082)	Percent	Controls (*n* = 50,031)	Percent	Crude RR (95% CI)
Sex					
Male	1,054	50.62	26,035	52.04	0.95 (0.87–1.03)
Female	1,028	49.38	23,996	47.96	1.00
Maternal age (years)					
< 20	28	1.34	292	0.58	2.34 (1.61–3.42)
20–24	215	10.33	5,545	11.08	1.00
25–29	892	42.84	24,281	48.53	0.95 (0.82–1.10)
≥30	947	45.49	19,913	39.81	1.22 (1.05–1.41)
Maternal education (years)					
< 9	16	0.77	270	0.54	1.47 (0.91–2.37)
9–12	59	2.83	1,332	2.66	1.11 (0.86–1.44)
12–16	1,281	61.53	32,330	64.62	1.00
≥16	726	34.87	16,099	32.18	1.13 (1.04–1.24)
Paternal education (years)					
< 9	28	1.34	414	0.83	1.76 (1.22–2.53)
9–12	108	5.19	1,703	3.40	1.65 (1.36–2.01)
12–16	1,135	54.51	26,257	52.48	1.15 (1.05–1.25)
≥16	811	38.95	21,657	43.29	1.00
Season					
Jan–Feb 2001	238	11.43	5,064	10.12	1.22 (1.03–1.45)
Mar–May 2001	287	13.78	7,067	14.13	1.06 (0.91–1.25)
Jun–Aug 2001	237	11.38	6,160	12.31	1.01 (0.85–1.20)
Sep–Nov 2001	262	12.58	6,420	12.83	1.07 (0.91–1.26)
Dec 2001–Feb 2002	280	13.45	6,558	13.11	1.12 (0.95–1.32)
Mar–May 2002	259	12.44	6,166	12.32	1.10 (0.93–1.30)
Jun–Aug 2002	246	11.82	5,421	10.84	1.18 (1.00–1.40)
Sep–Dec 2002	273	13.11	7,175	14.34	1.00

**Table 3:** Crude and adjusted RRs (95% CIs) of PTD attributable to maternal exposure to PM_10_, CO, SO_2_, and NO_2_ during the first trimester of pregnancy.

Pollutant	Exposure level	Crude RR (95% CI)	Adjusted RR (95% CI)^a^	Trend^b^
CO (mg/m^3^)	0.91–1.27	1.20 (1.06–1.34)	1.26 (1.11–1.44)	< 0.001
	0.78–0.90	1.10 (0.97–1.24)	1.14 (1.01–1.29)	
	0.64–0.77	0.92 (0.81–1.04)	0.92 (0.81–1.05)	
	0.47–0.63	1.00	1.00	
PM_10_ (μg/m^3^)	64.57–106.39	1.07 (0.95–1.21)	1.27 (1.04–1.56)	0.39
	53.84–64.56	1.02 (0.90–1.15)	1.13 (0.94–1.37)	
	45.95–53.83	1.06 (0.94–1.20)	1.14 (0.97–1.34)	
	26.99–45.94	1.00	1.00	
NO_2_ (μg/m^3^)	56.22–80.58	1.17 (1.04–1.34)	1.24 (1.09–1.41)	0.02
	43.12–56.21	1.09 (0.96–1.23)	1.07 (0.94–1.21)	
	29.68–43.11	1.14 (1.01–1.29)	1.13 (0.99–1.27)	
	10.41–29.67	1.00	1.00	
SO_2_ (μg/m^3^)	45.86–103.96	1.16 (1.03–1.31)	1.21 (1.04–1.42)	0.02
	22.75–45.85	1.09 (0.97–1.23)	1.13 (0.98–1.30)	
	17.62–22.74	1.11 (0.99–1.26)	1.13 (0.99–1.28)	
	7.86–17.61	1.00	1.00	

a Adjusted for maternal age, parity, sex, season of birth, and education level of father and mother.

b *p*-Value for the trend of adjusted RRs.

**Table 4:** Crude and adjusted RRs (95% CIs) of PTD attributable to maternal exposure to PM_10_, CO, SO_2_, and NO_2_ during third trimester of pregnancy.

Pollutant	Exposure level	Crude RR (95% CI)	Adjusted RR (95% CI)^a^	Trend^b^
CO (mg/m^3^)	0.88–1.16	1.14 (1.01–1.28)	1.16 (1.01–1.34)	0.03
	0.75–0.87	1.03 (0.91–1.16)	1.07 (0.94–1.22)	
	0.64–0.74	1.05 (0.93–1.18)	1.07 (0.95–1.21)	
	0.49–0.63	1.00	1.00	
PM_10_ (μg/m^3^)	65.63–95.91	1.06 (0.94–1.20)	1.09 (0.91–1.30)	0.33
	56.07–65.62	1.06 (0.94–1.19)	1.04 (0.90–1.21)	
	47.07–56.06	1.05 (0.93–1.18)	1.05 (0.91–1.20)	
	33.12–47.06	1.00	1.00	
NO_2_ (μg/m^3^)	57.67–76.12	1.22 (1.08–1.37)	1.21 (1.07–1.37)	< 0.001
	46.92–57.66	1.12 (0.99–1.27)	1.14 (1.01–1.29)	
	29.94–46.91	1.04 (0.92–1.18)	1.06 (0.93–1.20)	
	11.92–29.93	1.00	1.00	
SO_2_ (μg/m^3^)	46.54–103.15	1.04 (0.93–1.17)	1.11 (0.94–1.31)	0.26
	25.63–46.53	0.95 (0.84–1.07)	0.97 (0.83–1.13)	
	17.04–25.62	0.87 (0.77–1.02)	0.87 (0.76–1.01)	
	6.55–17.03	1.00	1.00	

a Adjusted for maternal age, parity, sex, season of birth, and education level of father and mother.

b *p*-Value for the trend of adjusted RRs.
